# Using symmetry to control viscoelastic waves in pillar arrays†

**DOI:** 10.1039/d3ra06565k

**Published:** 2023-10-27

**Authors:** Jason P. Beech, Oskar E. Ström, Enrico Turato, Jonas O. Tegenfeldt

**Affiliations:** a Division of Solid State Physics, Department of Physics, Lund University, Nano-Lund, Lund University PO Box 118 SE-221 00 Lund Sweden jonas.tegenfeldt@ftf.lth.se +46 46 222 8063

## Abstract

Solutions of macromolecules exhibit viscoelastic properties and unlike Newtonian fluids, they may break time-reversal symmetry at low Reynolds numbers resulting in elastic turbulence. Furthermore, under some conditions, instead of the chaotic turbulence, the result is large-scale waves in the form of cyclic spatial and temporal concentration variations, as has been shown for macromolecular DNA flowing in microfluidic pillar arrays. We here demonstrate how altering the symmetry of the individual pillars can be used to influence the symmetry of these waves. We control the extent of instabilities in viscoelastic flow by leveraging the effects of the symmetry of the pillars on the waves, demonstrating suppressed viscoelastic fluctuations with relevance for transport and sorting applications, or conversely opening up for enhanced viscoelasticity-mediated mixing. The onset of waves, which changes flow resistance, occurs at different Deborah numbers for flow in different directions through the array of triangular pillars, thus breaking the symmetry of the flow resistance along the device, opening up for using the occurrence of the waves to construct a fluidic diode.

## Abstract 2

Leveraging symmetry of pillar arrays to control instabilities and mixing of high-concentration DNA solutions.

## Graphical abstract

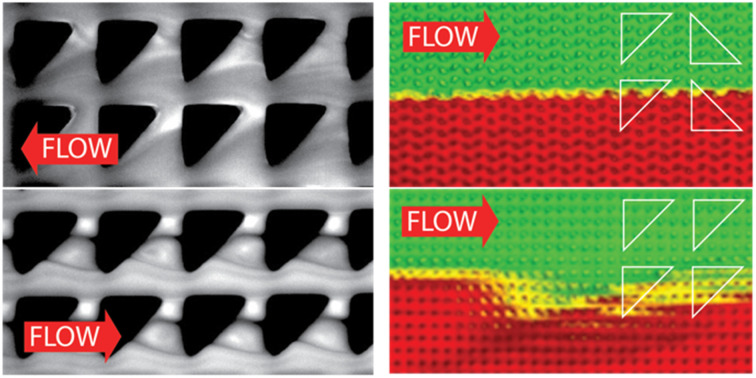

## 1 Introduction

*Viscoelastic effects* in the flows of polymer solutions through and around structures at the microscale are believed to have practical importance in diverse areas ranging from the extraction of oil from mineral matrices^1^ and the transport of heat,^2^ to the understanding of the transport of biological fluids, both in their native environments^3^ and through novel types of microfluidic devices.^4,5^ An example of the latter is microfluidics devices for the separation of DNA molecules by length that use deterministic lateral displacement (DLD)^6–11^ as well those relying on pulsed electrical fields combined with a pillar array arranged in a hexagonal pattern.^12,13^ While the underlying mechanisms are entirely different, both are based on arrays of pillars through which the molecules follow size-dependent trajectories. Since solutions of DNA are viscoelastic fluids, and therefore may exhibit elastic turbulence,^14,15^ an understanding of their behavior in pillar arrays is essential for the development and improvement in functionality of these devices.

The fluctuations and instabilities that viscoelastic flows exhibit have been observed for a broad range of geometries.^16–18^ Indeed, during recent efforts to improve the separation of long (>20 kbp) DNA fragments in DLD devices^11^ we found that DNA can assemble into ordered waves of high concentration and molecular stretching and, what is more, that the local flow direction inside these waves differs from the bulk flow direction, an effect which both increased and decreased the quality of separation dependent on the specific conditions. We have subsequently shown that the formation of these waves depends to a large degree on the viscoelastic properties of the fluid and the order of the pillar array, with large scale flow patterns or waves absent in arrays where the position of each pillar has been randomly perturbed.^19^

*Symmetry* plays an important role in this context. DLD is by its nature asymmetric. Particles move across flow lines due to steric interactions between the particles and the pillars, something that was first observed to be essential for sorting in early ratchet devices,^20^ and which leads to the breaking of the time-reversal symmetry of low-Reynolds number flow in DLD devices.^21^ Furthermore, the geometry of the pillar array and device boundaries may introduce a lateral flow component,^22^ an effect that can be somewhat mitigated by the addition of carefully designed compensatory structures to the array boundaries.^23,24^ For DLD, decreasing the symmetry of the cross section of pillars from circular to triangular^21,25^ has been used to decrease the critical size, the propensity for clogging and to increase the sensitivity to deformability when sorting cells.^26^ In viscoelastic flows, longitudinal symmetry is broken by deadzones forming upstream of pillars.^17^ Lateral symmetry has been shown to be spontaneously broken for flow around a cylindrical laterally symmetrical obstacle.^27–30^ Symmetry plays an important role for instabilities in arrays of symmetric (circular and square)^17^ and non-symmetric (triangular)^31^ pillars.

The *instabilities* that readily occur even at low Reynolds numbers for viscoelastic fluids offer opportunities as well as challenges.^15^ They may influence throughput through changes in flow resistance. They are detrimental to the integrity of sample streams and sample plugs. On the other hand, for mixing applications they offer an excellent substitute for inertial turbulence that is difficult to achieve in microfluidic systems. There is thus a strong interest in understanding the origin of the instabilities as well as in the development of strategies for controlling them. While randomization of the pillar structure has been shown to suppress the instabilities,^19,32^ for certain cases a randomization can enhance instabilities,^33^ and for some applications it may be unsuitable where a regular array is required for optimal operation.^7,13^ Other approaches for suppression involve careful channel design,^34–36^ soft borders^37^ and flow modulation.^38^

*Mixing* is a central function in many microfluidic systems,^39,40^ for example in biological assays, medical analysis, material synthesis, heat transfer and the study of chemical reactions where it is essential for accurate and reproducible results.^40^ Mixing is a process that leads to uniformity of concentration and on a fundamental level it relies on diffusion. Due to the quadratic dependence of diffusion time on diffusion length, in most cases various approaches are necessary to decrease the diffusion length in order to reach acceptable sample processing times.^41^ In the macroscopic world, turbulence will do this simply by stirring. In microfluidics, more elaborate schemes are necessary to shape the flow of the liquids so that the different samples are folded into each other.^42^ At the low Reynold's numbers ubiquitous in microfluidics devices, viscoelastic instabilities and viscoelastic turbulence have been shown to increase mixing.^4,43^

*Diodicity* may be the consequence of broken device symmetry. The difference in flow resistances in the two flow directions in a device can be traced back to different flow patterns and has been demonstrated for both Newtonian^44^ and viscoelastic systems.^31,45^ Under low-Reynolds number conditions, viscoelastic fluids are of especial interest for these types of applications.

In this paper we explore the role of the symmetry of the array pillars on the macroscopic waves that we have reported previously.^19^ We then use what we have learnt to show proof of principle of prospective applications in terms of stabilized flows, mixing and rectified flows.

## 2 Materials and methods

In the following, a brief overview is given about the experimental procedures as well as a discussion about dimensionless numbers that are used in the analysis. Further details are described in the ESI Sections 1 to 6 and 8.†

### 2.1 Reagents

*λ* phage DNA (New England Biolabs, Ipswich, MA, USA) at a concentration of 400 μg ml^−1^ was stained with the bisintercalating dyes YOYO-1 (green) or YOYO-3 (red) (Life Technologies, Carlsbad, CA, USA). YOYO-1 was used for the main studies of symmetry, and two samples with each of the dyes were used for the demonstration of mixing and suppression of mixing. For the two-color imaging, the staining ratios were adjusted to take into account the difference in brightness of the two dyes.

### 2.2 Devices

Three different device designs were used in our study. The devices were fabricated using standard replica molding techniques with a design based on a straight channel with a square array of pillars of overall size 0.8 mm wide, 8 mm long, and 11.5 μm deep. All devices have a pitch of 36 μm and an array consisting of 22 × 222 triangles.

**Fig. 1:**
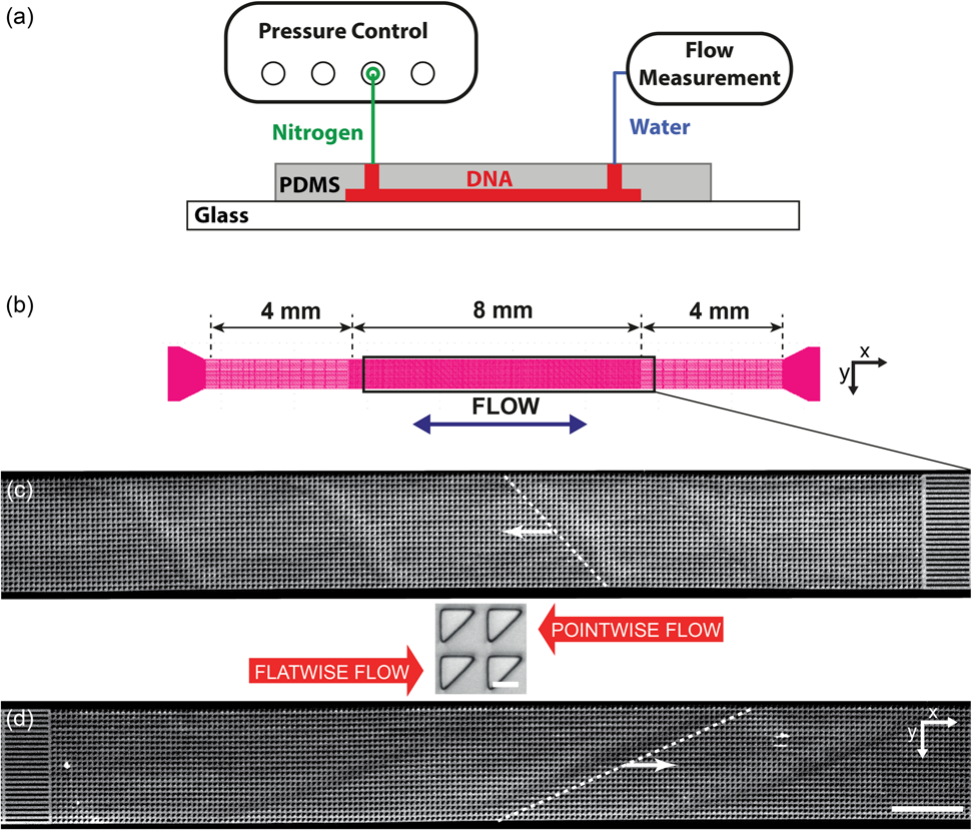
Overview of the micropillar platform and snapshots of fluorescent DNA moving through an array of triangular pillars (device 1). (a) Schematic of setup. Note that the measurement takes place using water for which the flow sensor has been calibrated. The DNA does not reach the flow sensor. (b) Device layout with the region of interest indicated in the center. Note that the region of interest is approximate. It is adjusted such that the straight channels at the beginning of the flow are included in the field of view for each flow direction. (c and d) Low-magnification micrographs (2×) for the two flow directions. In each case, one wave is highlighted with a dashed white line. (c) Pointwise flow (*u* ≈ 8.7 mm s^−1^, De ≈ 350). (d) Flatwise flow (*u* ≈ 10 mm s^−1^, De ≈ 400). The dynamics of the waves is clearly seen in the ESI Movies S1, S4, and S5.† The intensity and brightness are the same for all figure panels and set to enhance the visibility of the waves (as for all subsequent figures). Scale bar 500 μm for the low-magnification snapshots and 20 μm for the inset showing the pillars.

For the main work on the effect of symmetry, device 1 is used. It consists of a straight channel with one inlet and one outlet. The array consists of right angled isosceles triangular pillars, see Fig. 1(c), (d) and S1 in the ESI.† The legs of the triangles are 27 μm and the gaps between them are 9 μm. The pillars are oriented such that flow in one direction impinges on a leg (which we denote flatwise flow), and in the other direction on an acute vertex (which we denote pointwise flow).

For the wave-suppression studies and the mixing studies, the devices have two inlets and one outlet, with two types of arrays: device 2 with triangular pillars in the flatwise orientation but with each row of alternating handedness (left or right), and device 3 with right angled isosceles triangular pillars in the flatwise orientation just like device 1, see Fig. 5(a) and S2 in the ESI.† For device 2 the two equal sides of the posts are 25 μm and the gaps 10 μm. For device 3 the two equal sides of the posts are 23 μm and the gaps 12 μm. The differences in post size originate in the fabrication processes.

### 2.3 Experimental setup

The devices were run by applying an overpressure of nitrogen gas, and the flow rate measured using an inline liquid flow sensor (Fluigent, Paris, France).

The DNA was imaged using standard epifluorescence microscopy. We used a 4× (Nikon Plan Apo *λ*, NA 0.2) and a 40× (Nikon Plan Fluor, NA 0.60) objective together with a sCMOS Hamamatsu Orca Flash 4.0 camera (Hamamatsu, Shizuoka Pref., Japan) to acquire raw images.

For the two-color imaging, an Optosplit II (Cairn Research Ltd., Faversham, UK) was used for simultaneous acquisition of the two emission wavelengths coming from the YOYO-1 and YOYO-3 stained DNA subpopulations.

Any confounding patterns caused by diffraction or reflection in the array were characterized for device 1 without DNA (see Section 6 in the ESI†). Image processing was done with custom made Python code using standard libraries.

### 2.4 Dimensionless numbers

To be able to relate our observations to the prevailing flow conditions we derive the average flow velocities from the measured volumetric flow rates, *Q*, 0.01–8 nl s^−1^, dividing by the total cross-sectional area of the smallest gaps between the pillars, *A*. We estimate the Reynolds number, Re [eqn (1)], for all experiments to be in the range 9 × 10^−5^ to 6 × 10^−2^, such that we can neglect any inertial contributions. Here *ρ* is the density of water, *G* is the gap between the pillars, and *μ* is the viscosity measured as described in the ESI Section 4.†1
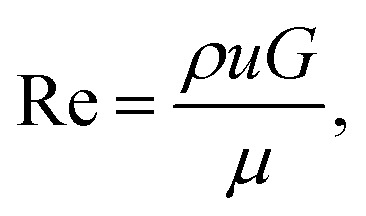


The Deborah number is evaluated using eqn (2):2
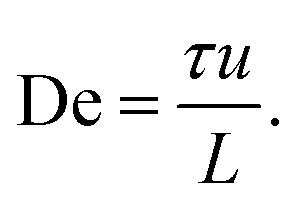


Here *τ* = 1.43 s is the relaxation time for *λ* DNA (measurement detailed in ref. 19), and *L* = 36 μm is the array pitch. *u* is the mean flow velocity between pillars, see detailed description of *u* in the ESI Section 4† resulting in a De in the range 1.4–400. Elastic effects can thus not be neglected.

These dimensionless numbers are calculated using estimates of the mean flow rates between pillars and using the bulk properties of the DNA solution and should be considered as nominal values and guides for comparison only since the presence of vortices and the variation in the local concentration of DNA (as easily seen in Fig. 3) lead to large variations in these numbers in time and space. What is more, the measured flow rates for the given applied driving pressures differ between the flatwise and pointwise directions, leading to different De (see discussion on diodicity below).

## 3 Results and discussion

As a solution of *λ* phage DNA at high concentration exceeding the overlap concentration as in our previous work^19^ is forced to flow through the pillar array at various applied pressures, the fluorescence images give us information about the local concentration of the DNA as it varies over space and time. We observe several phenomena with large qualitative differences immediately apparent in the two flow directions. We consider first macroscopic observations of concentration waves over the entire device and will then continue to discuss the flow at the scale of individual posts. Finally, we show proof of principle of prospective applications. Additional experimental results for a broader range of experimental parameters are shown in the ESI Sections 6 to 8 along with descriptions of movies in Section 9.†

### 3.1 Macroscopic waves

In Fig. 1 we can see how the symmetry of the macroscopic wave pattern is broken for the array with triangular pillars. As opposed to the case of circular pillars which generates two types of waves with orientations mirrored around the axis along the device channel with equal probability, as previously reported,^19^ only one type of wave dominates for each flow direction when we break the lateral symmetry using the triangular pillars. This is in contrast to the spontaneous lateral symmetry breaking around circular obstacles that lacks any preferential direction.^27–30^ Note that there is a clear difference in the magnitude of the angles with respect to the device axis of the orientations of the two waves, implying that the underlying mechanisms are different.

**Fig. 2:**
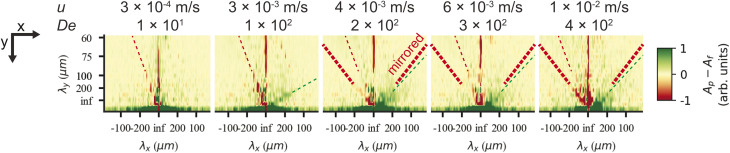
Low frequency region of spatial Fourier amplitude difference spectra for pointwise (green) minus flatwise (red) flows in device 1. Before subtraction, the mean spectra for all the frames of each videograph were computed. The data is based on fluorescence videos captured at a low magnification (2× objective) of various lengths in the range of 100 to 300 s. For clarity and to better compare with the relevant scales, instead of giving the inverse distances corresponding to each Fourier component, we give the lengths. Guides for the eye are added as follows. The thin red dashed line (all panels) corresponds to an optical artefact (see Section 6 in the ESI†). The thick red dashed line corresponds to waves for flatwise flow (mirrored for comparison with pointwise waves). The dashed green line corresponds to waves for pointwise flow. Note that pointwise waves occur at lower De (De = 100 for pointwise waves compared to De = 200 for flatwise waves). See ESI, Section 8† for further discussion about the frequency analysis and for additional data.

Analyzing the spatial, two-dimensional (2D) frequency components of the macroscopic wave patterns in the DNA seen in Fig. 1 reveals a distinct difference in the Fourier transform amplitude spectra for the two flow directions for flow velocities above approximately 3 × 10^−3^ m s^−1^, see Fig. 2 for the low-frequency components. The waves corresponding to the pointwise flow (green) appear for lower flow velocities than the waves corresponding to the flatwise flow (red). The angles corresponding to a high amplitude for each flow direction in the high velocity spectra (3 × 10^−3^ m s^−1^, 6 × 10^−3^ m s^−1^, and 1 × 10^−2^ m s^−1^) correspond to the orientations of the waves of the two directions visible in Fig. 1. Note that the difference is not as clear as in the images, presumably because the angle of the waves flatten out close to the walls in pointwise flow.

**Fig. 3:**
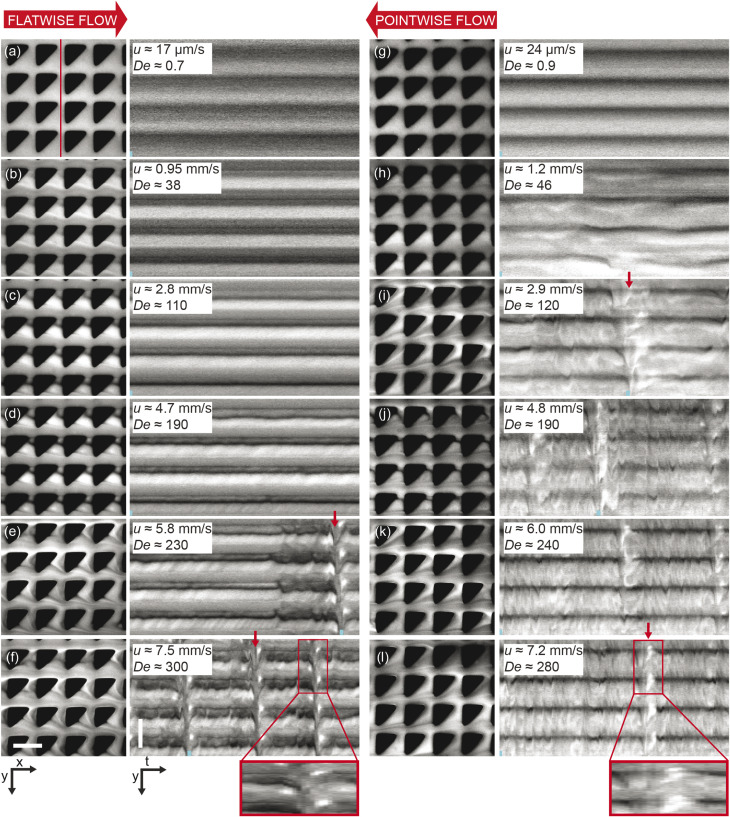
Microscopic flow patterns for device 1. Observed at the microscale (40× magnification), the flow behavior differs significantly between the two flow directions. The pairs of images in each panel consist of micrographs (*x*, *y*) and kymographs (*y*, *t*) taken along the vertical line shown in the first panel. The small blue bars found at the bottom of each kymograph indicate the relative time points of the micrograph in each pair. The red arrows highlight a selection of the time points when waves occur. Each kymograph spans a total of 7 s. To illustrate the evolution of the high-concentration DNA blobs, regions of interest spanning 0.9 s and expanded in the time dimension are indicated in (f) and (l) at the very bottom. See the ESI Movie S2† corresponding to the data of all the panels. Scale bars are 20 μm.

We can estimate a threshold value for De for which macroscopic waves form from Fig. 2 noting that a clear signal is visible for De ≥ 200 for flatwise flow and De ≥ 100 for pointwise flow. On the other hand, by observation in the raw data presented in the ESI Fig. S5 and S6,† we instead make the estimates De ≥ 140 for flatwise flow and De ≥ 100 for pointwise flow. For the corresponding Fourier transforms in Fig. S8 and S9,† we make the estimates De ≥ 170 for flatwise flow and De ≥ 100 for pointwise flow.

See ESI Fig. S5 and S6† for low-magnification micrographs of a larger range of flow velocities and Fig. S8 and S9† for amplitude spectra of the full frequency range.

### 3.2 Microscopic flow patterns

On the microscopic scale of the pillars, for flow in both directions, as the flow velocity is increased from zero, the first observation we make is that stable deadzones depleted of DNA form and that their distributions differ between the two directions, Fig. 3(a) and (g). This corresponds to what has been reported in viscoelastic flows in pillar arrays of different shapes,^17,31^ and is similar to what we see for circular pillars.^19^ As the flow velocity is increased, the asymmetry becomes more pronounced. Vortices form, similar to ref. 46–49, but their positions, strength, fluctuations, and interactions with each other are different for the two flow directions, see Fig. 3(b)–(d) and (h). Finally, as can be seen in Fig. 1 and 3(e), (f) and (i)–(l), waves form that are very different on both the microscopic and macroscopic scale.

**Fig. 4:**
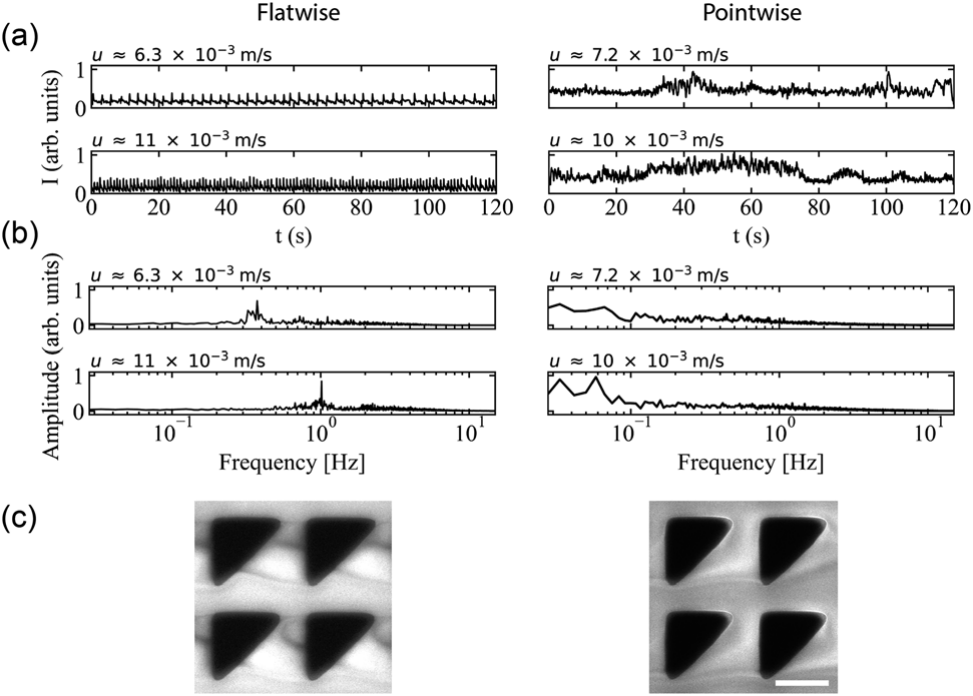
Microscopic concentration fluctuations around individual pillars for flatwise and pointwise flows in device 1. (a) Temporal fluctuations in intensity around one pillar as indicated in Fig. S3(c), ESI.† (b) Corresponding temporal Fourier amplitudes. (c) Flow characteristics around four pillars in flatwise (left) and pointwise flow (right) (20× magnification) from ESI Movie S3.† Scale bar is 20 μm.

Flow in the *flatwise direction* is dominated by the formation of distinct non-symmetric vortex pairs between pillars along the flow direction at high De, with a smaller clock-wise vortex near the vertex of the pillar and a larger counter clock-wise brighter vortex below, see Fig. 4(c), consistent with the abrupt increase in vorticity observed for the viscoelastic flow in a channel with a pair of cylindrical obstacles.^46^ The formation of these vortex pairs can be clearly seen in Fig. 3(d) at *u* = 4.7 mm s^−1^ and they persist between waves at higher flow rates, Fig. 3(e) and (f). Immediately prior to the passage of a wave the vortices appear to entangle and subsequently mix and vanish, resembling the cycles of buildup of a deadzone followed by deadzone washing as reported in ref. 17 although in their case the observation is not connected to any device-wide regular fluctuations. Our waves are associated with a fairly abrupt accumulation of DNA in the vortices as is clear from the inset in Fig. 3(f).

**Fig. 5:**
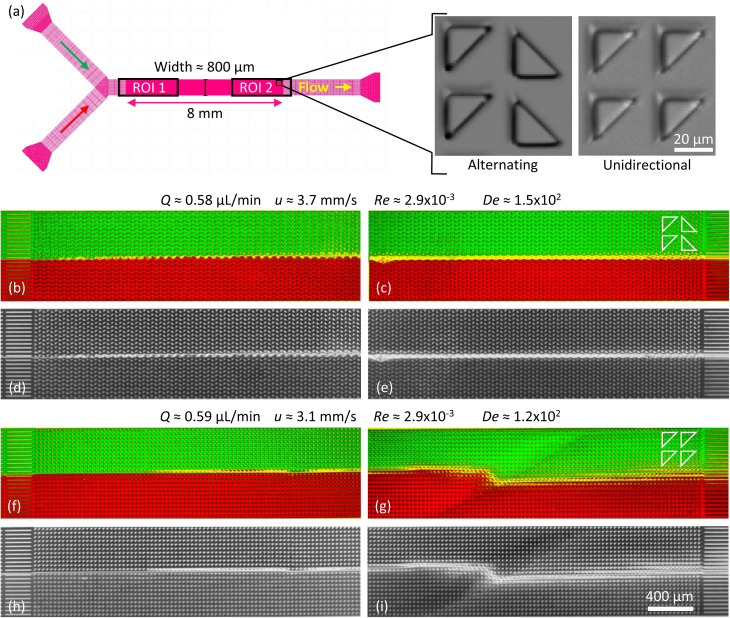
Suppression of viscoelastic fluctuations and enhancement of mixing in devices 2 and 3 for low flow rates. (a) Device layout for device 2 (alternating) and device 3 (unidirectional). (b–i) Two DNA solutions stained in red and green respectively, are introduced in the inlets at the left of the device. The left panels refer to the upstream part of the device (ROI 1) and the right panels refer to the downstream part of the device (ROI 2). The overlap is calculated as the smallest of the ratios of the two colors and is shown in yellow. The gray-scale images below each color image show the overlap ratio only. (b–e) Arrays of triangles with a periodical alternating orientation resulting in a clear suppression of the waves. (f–i) Arrays of triangles with unidirectional orientation leading to clear wave formation. Note that the array geometry is indicated using the white outlines of the pillars to the right for each case.

In the *pointwise direction*, the evolution of the flow pattern is not as clear cut as for the flatwise flow. Stable vortices are not observed, see Fig. 4(c). Instead, the flow of DNA exhibits two distinct phenomena: rather stable, yet minuscule wakes form at each downstream vertex during the period between waves resembling those described, for example, in ref. 17 followed by the wakes vanishing as the wave of high-concentration DNA passes. In contrast to the flatwise direction, here waves are associated with a gradual shift of the accumulated DNA as is seen in the inset in Fig. 3(l).

An analysis of the intensity in a unit cell of the array around one pillar further highlights the differences in flow behaviour in the two flow directions. Fig. 4(a) shows a striking difference in the time evolution of the intensities, *i.e.* local DNA concentrations, around a pillar due to the propagation of the wavefronts for the two flow directions in terms of the regularity of the fluctuations. A Fourier analysis of the time signals, see Fig. 4(b), identifies a distinct frequency peak (at approximately 1 Hz) for flatwise flow which is absent for pointwise flow. The difference in the dynamics may be ascribed to the presence of the wakes, or stagnation points, which have been identified as a source of instabilities.^33^

We can estimate a threshold value for De for which microscopic instabilities and waves form from Fig. 3 noting that for flatwise flow instabilities start at De = 190 and waves start at De = 230 and for pointwise flow instabilities start at De = 46 and waves start at De = 120. On the other hand, by observation of the fluctuations of the intensity around one pillar presented in the ESI, Fig. S10,† and the corresponding Fourier transform in Fig. S11,† we instead make the estimates for the onset of instabilities at De ≥ 230 for flatwise flow and De ≥ 150 for pointwise flow.

For a better view of the formation and shedding of vortices as well as the flow dynamics in general we refer to the ESI Movies S2 and S3.†

### 3.3 Applications

To demonstrate the technical relevance of our work, we show proof of principle of how the control of the waves can be useful for suppressing viscoelastic fluctuations for future transport and sorting applications, for enhanced mixing, and for fluid rectification. We also direct the reader's attention to relevant questions of the underlying physics that are necessary to understand in detail to fully optimize these applications.

To demonstrate *suppression of viscoelastic instabilities*, we selected a design based on flatwise flow combined with breaking the lateral symmetry with alternating left- and right-handed orientations of the triangles in each row (device 2), we indeed note that the waves are strongly suppressed, in turn also suppressing the mixing. The region of overlap is successfully minimized and constrained to a thin line in the middle of the device, almost limited to a single row of pillars. See Fig. 5(b)–(e). We argue that changing the orientation of the triangles in each row, neither the right-handed nor the left-handed wave has a chance to form, in analogy to the open-loop modulation demonstrated in simulations for suppression of instabilities.^38^ This suggests that the asymmetric waves are formed through an additive process such that each step leading up to a left-handed wave simply constitutes the inverse of the corresponding step for a right-handed wave. Further studies might shed light on how this contributes to the long-range coupling between the flow on the scale of the pillars to the waves that span the entire device. It also implies that the angle of the waves may be related to the balance between the axial flow of the liquid and the lateral gradual buildup of the conditions that lead to wave formation. The difference in angle of the waves for flatwise and pointwise flow directions that can be clearly seen in Fig. 1(c) and (d), together with the fact that the local flow patterns are so distinct, see Fig. 4(c), implies a significant connection between the microscopic flow patterns and the exact mechanism for the coupling between the short-range patterns on the scale of the pillars and the long-range wave patterns.

**Fig. 6:**
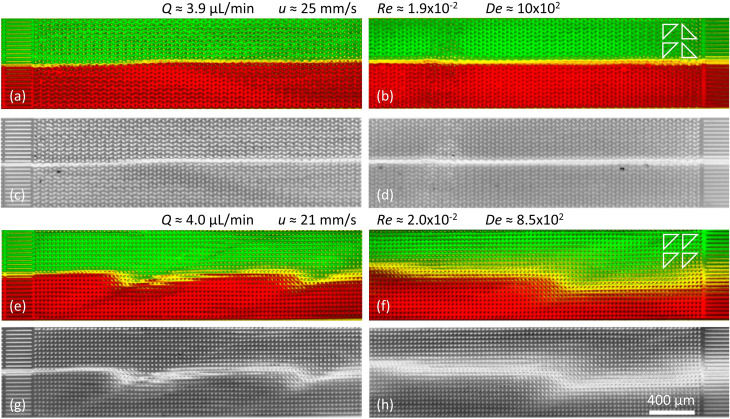
Suppression of viscoelastic fluctuations and enhancement of mixing in devices 2 and 3 just like in Fig. 5 but for high flow rates. (a–d) Arrays of triangles with a periodical alternating orientation resulting in a clear suppression of the waves. (e–h) Arrays of triangles with unidirectional orientation leading to clear wave formation and strong mixing.

It is important to mention that at high flow rates some viscoelastic instabilities emerge close to the inlet region for device 3, Fig. 6(a). These do not result in any lateral transport of the DNA, and thus not to any increased mixing. They seem to dissipate as they flow through the device, and cannot be seen at the outlet, Fig. 6(b). We argue that these waves are being generated at the inlet due to the transition from the straight channel to the obstacles arrays, but that the alternating triangle geometry efficiently suppresses them. For a better view of the waves and the mixing as well as the flow dynamics in general we refer to the ESI Movies S6 and S7.†

As a control we demonstrate *enhanced mixing*, by selecting a design based on flatwise flow with an array of triangles arranged similarly oriented in all rows (device 3). Feeding two streams of differently colored DNA side by side into the device, the resulting waves lead to enhanced overlap of the two DNA streams, Fig. 5(f)–(i) and 6(e)–(h). We see that the amount of waves increases with the flow rate. The waves give rise to a clear overlap of the two input streams and the mixing region is wider at higher flow rates. The extent of the overlap is on the order of three magnitudes larger than would be expected by diffusion alone, calculated based on the radius of gyration of the DNA and the measured average viscosity of the DNA solution.

**Fig. 7:**
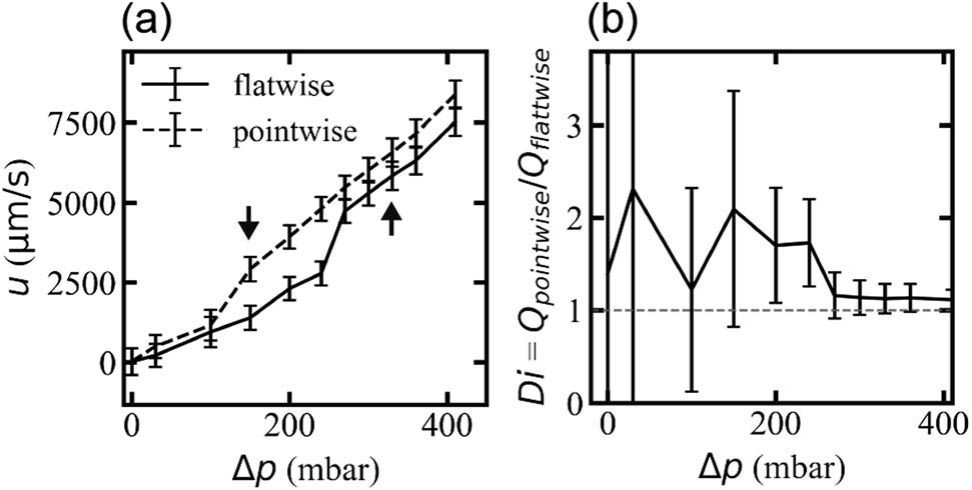
Different flow resistance for different flow directions for device 1. (a) Mean flow velocity dependence of applied pressure. (b) Diodicity, Di = *Q*_pointwise_/*Q*_flatwise_ as a function of applied pressure. The arrows in (a) mark the earliest wave observations in pointwise (150 mbar, left arrow) and flatwise (330 mbar, right arrow). The error bars in (a) represent one standard deviation and those in (b) represent the fractional standard deviations added in quadrature.

Flow rates differ for the two applied flow directions at each applied pressure for the array with triangles arranged similarly oriented in all rows (device 1), presumably due to the difference in microscopic flow patterns.^45^ However, what is more important for any application as a *fluidic diode* is that the flow resistance changes at the onset of waves and that waves appear for different pressures for the two directions. We ascribe this difference in threshold to stabilization of the flow by the strong vortices in the flatwise flow such that a higher applied pressure is necessary than for pointwise flow to give rise to waves. The difference in flow rate is thus maximized for the pressure range for which waves occur in only one of the two flow directions, Fig. 7. The ESI Movies S4 and S5† show this difference clearly during the application of a slowly cycled driving pressure.

## 4 Conclusions

The work constitutes a first step to detailed understanding of the wave phenomenon that would enable the engineering of these regular viscoelastic instabilities for a wide range of fluidics applications. We have demonstrated the suppression of viscoelastic instabilities in DNA solutions, which may open up for stabilized transport of viscoelastic solutions at high concentrations and high throughput and unperturbed particle sorting involving various biological fluids such as saliva^50,51^ and blood plasma.^52^ It will also help efforts to sort DNA at high concentration and high throughput.^11^ Care must be taken in this case, however, to take into account any effects of the orientation of the flow in relation to the pillar array as is necessary for DLD^7^ but has been shown to influence the viscoelastic instabilities.^33^ To make the control of the waves the basis for a generally applicable tool, specifically for mixing applications at low Re, for example for devices for immunocapture,^53^ it will be necessary to explore whether more generic molecules such as polyethylene glycol (PEG) can be used instead of DNA.

The concomitant symmetry breaking^54^ at the scale of the waves and at the scale of the pillars suggests that changes of the shape of the pillars may be used to modulate the characteristics of the waves, something that is also supported by the sharp difference in the microscopic fluctuations for the two flow directions. To verify this hypothesis, a systematic screening of possible pillar shapes is necessary. Intuitively, the waves are no surprise due to the feedback between the flow through the pillar array causing the waves, and the waves influencing the flow by their higher local viscosity. For detailed understanding of the underlying mechanisms, careful high-speed flow trajectory measurements will be necessary, *e.g.* using particle tracking velocimetry,^55^ especially during the passage of a wave across a pillar on microscopic as well as macroscopic scales. During the entanglement of the vortices, the flow normal to the device is especially interesting, making it necessary to explore the effect of varying the depth of the device. A central question is how the microscopic behavior around each pillar is synchronized across the device to form the macroscopic waves. The results of the alternating triangular pillar arrays, may possibly be used as a basis for addressing this question using carefully designed combinations of different types of pillar geometries so that the contribution of different types of microscopic flow patterns to the generation of the macroscopic waves can be elucidated. Also, the various length scales in the array are important and need to be investigated. For example, changing the gap between the posts and the rows would allow us to characterize the relevant interaction distances, above which macroscopic waves would not be expected. Simulations will be required although they will not be trivial due to the large range of relevant length scales involved from μm in the vortices and other flow patterns close to each pillar to the device-wide waves on the mm scale.

The observation that the break in symmetry of the device design creates a difference in onset of wave formation, suggests a potential application of the observed flow phenomena as the basis for a fluidic rectifier.^45^ To optimize the operation of these types of devices, careful measurements of thresholds in terms of De will be needed including identifying any hysteresis.

## Data availability

The data that support the findings of this study are openly available in Harvard Dataverse at https://doi.org/10.7910/DVN/RY7MU8.

## Author contributions

The author contribution statement has been written according to CRediT (Contributor Roles Taxonomy, see https://credit.niso.org for role descriptions): conceptualization, J. P. B., O. E. S., E. T., and J. O. T.; data curation, J. P. B., O. E. S., and E. T.; formal analysis, J. P. B., O. E. S., and E. T.; funding acquisition, J. O. T.; investigation, J. P. B., O. E. S. and E. T.; methodology, J. P. B., O. E. S., E. T., and J. O. T.; project administration, J. O. T.; resources, J. O. T.; software, J. P. B., O. E. S., and E. T.; supervision, J. P. B., and J. O. T.; validation, J. P. B., O. E. S., E. T., and J. O. T.; visualization, J. P. B., O. E. S., E. T., and J. O. T.; writing – original draft, J. P. B., O. E. S., and J. O. T.; writing – reviewing & editing, J. P. B., O. E. S., E. T., and J. O. T. At a late stage of the project, during the preparation of the manuscript, we decided to include applications of symmetry for applications of mixing and suppression of mixing. This work constitutes the main contribution of E. T. in terms of experiments, data processing and analysis.

## Conflicts of interest

There are no conflicts to declare.

## Supplementary Material

RA-013-D3RA06565K-s001

RA-013-D3RA06565K-s002
