# Supplementary material for: Using symmetry to control viscoelastic waves in pillar arrays
Source: RSC Adv. 2023 Oct 27;13(45):31497–506. doi: 10.1039/d3ra06565k (PMC10603618; doi:10.1039/d3ra06565k)
Supplement: RA-013-D3RA06565K-s001 [file RA-013-D3RA06565K-s001.pdf]

Electronic Supplementary Material (ESI) for RSC Advances.
This journal is © The Royal Society of Chemistry 2023

Supplemental Material for

Using symmetry to control viscoelastic waves in pillar arrays

Jason P. Beech, Oskar E. Ström, Enrico Turato, and Jonas O. Tegenfeldt

(Dated: October 11, 2023)

1. DEVICE DESIGNS

Three different devices were made for the symmetry experiments (device 1), the demonstration of suppression of waves and mixing (device 2), and the demonstration of mixing (device 3). See Fig. S1 for device 1 and Fig. S2 for devices 2 and 3.

The devices were fabricated using standard replica molding techniques with a design based on a straight channel with a square array of pillars of overall size 0.8 mm wide, 8 mm long, and 11.5 µm deep. All devices have an array consisting of 22 × 222 right angled isosceles triangular pillars with a pitch of 36 µm, as can be seen in Fig. S1. The design parameters are the same for the overall dimensions as well as for the pillar dimensions for each device. However, due to variation in conditions during microfabrication, the actual dimensions deviate slightly between the different devices as described below.

Device 1 - The pillars are oriented such that flow in one direction impinges on a leg (which we call flatwise flow), and in the other direction on an acute vertex (which we call pointwise flow). For device 1 the two equal sides of the posts are 26.6 ± 0.6 μm and the gaps 8.7 ± 0.6 μm.

Device 2 and 3 - For the wave-suppression and mixing studies the devices have two inlets and one outlet, with two types of arrays: device 2 with triangular pillars in the flatwise orientation but with each row of alternating handedness (left or right), and device 3 with right angled isosceles triangular pillars in the flatwise orientation as device 1, see Fig. S2. For device 2 the two equal sides of the posts are 25.0 ± 0.8 μm and the gaps 10.3 ± 0.7 μm. For device 3 the two equal sides of the posts are 23.1 ± 0.5 μm and the gaps 12.4 ± 0.6 μm.

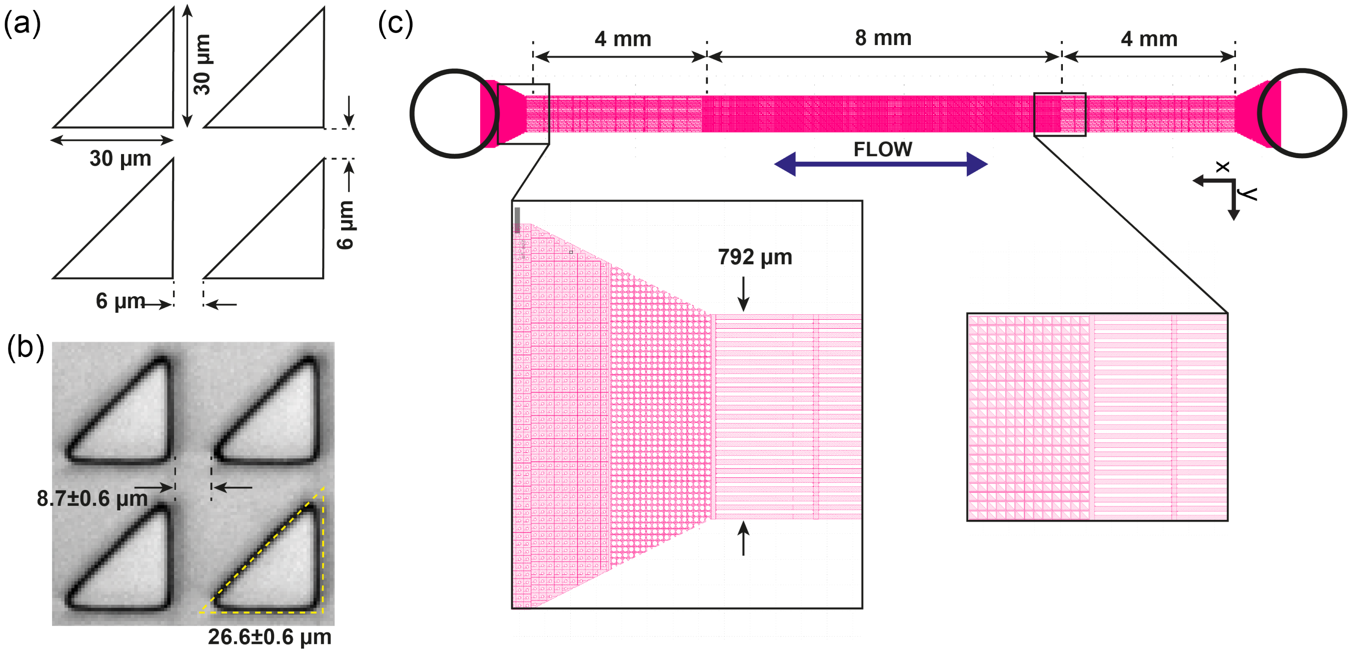

FIG. S1. Overview of the device 1. (a) Pillars were designed as right isosceles triangles. (b) Replica molded pillars in polydimethylsiloxane (PDMS). The PDMS pillars have slightly rounded corners giving a larger gap than in the design files. (c) The device consists of a channel containing an array of right-angled triangular pillars (22 pillars wide by 222 long) shown in red. To minimize any perturbations due to the proximity to the reservoirs, straight parallel channels are defined at each end.

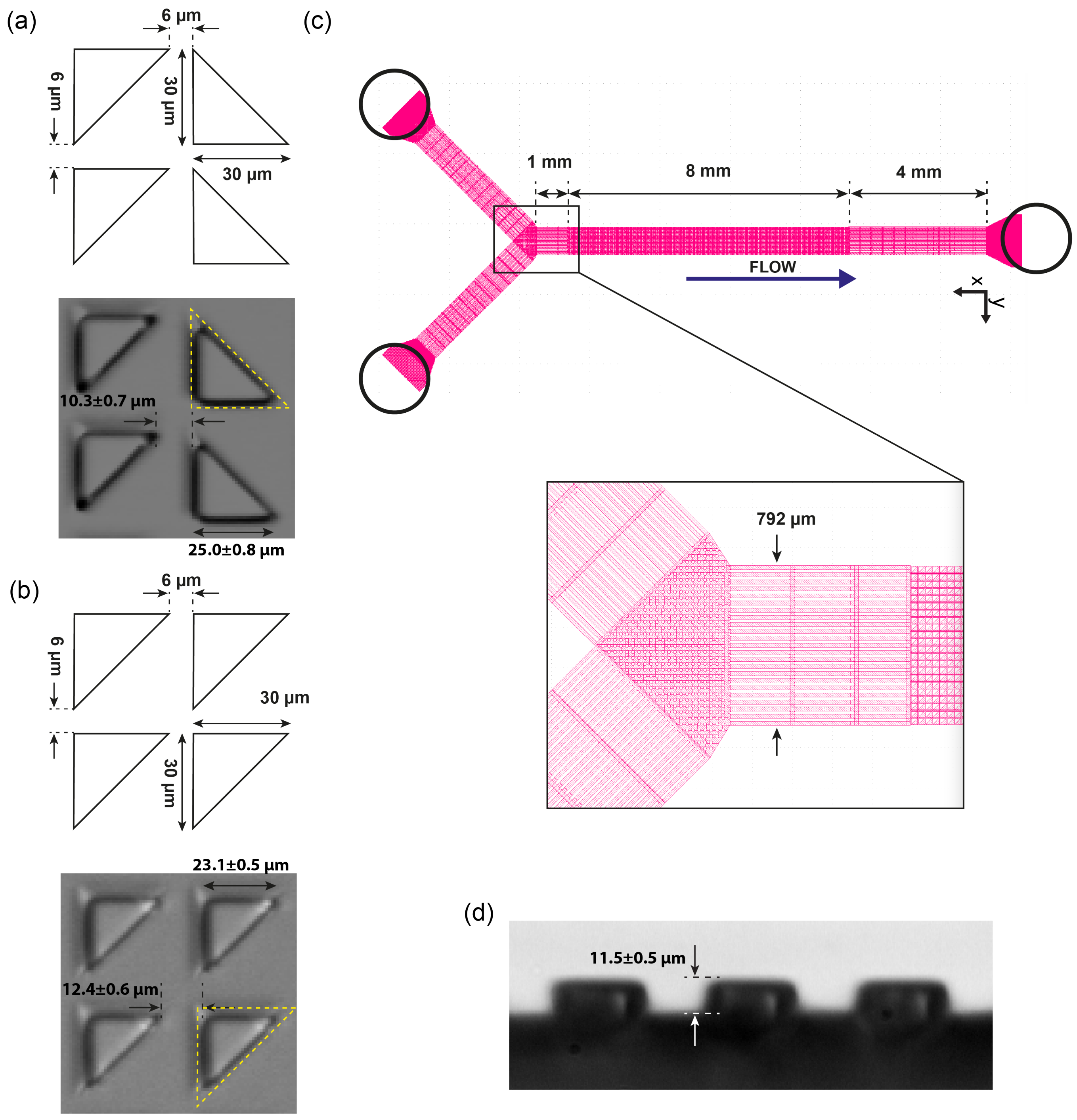

FIG. S2. Overview of the devices 2 and 3. (a) Device 2. Pillars designed as right angled isosceles triangular pillars in the flatwise orientation alternating in their lateral symmetry. (b) Device 3. Pillars designed as right angled isosceles triangular pillars in the flatwise orientation as device 1, see Fig. S1. (a, b) Bottom: Replica molded pillars in polydimethylsiloxane (PDMS). (c) The structure of the microfluidic channel shared by both device 2 and device 3. The devices have a similar structure to Fig. S1(c) but this time they consist of two inlets and one outlet. Notice that, as before, to minimize any perturbations due to the proximity to the reservoirs, straight parallel channels are defined at each end. (d) The height of the pillars, and thus the dept of the channel, is shown here. Notice that this image is representative for all devices.

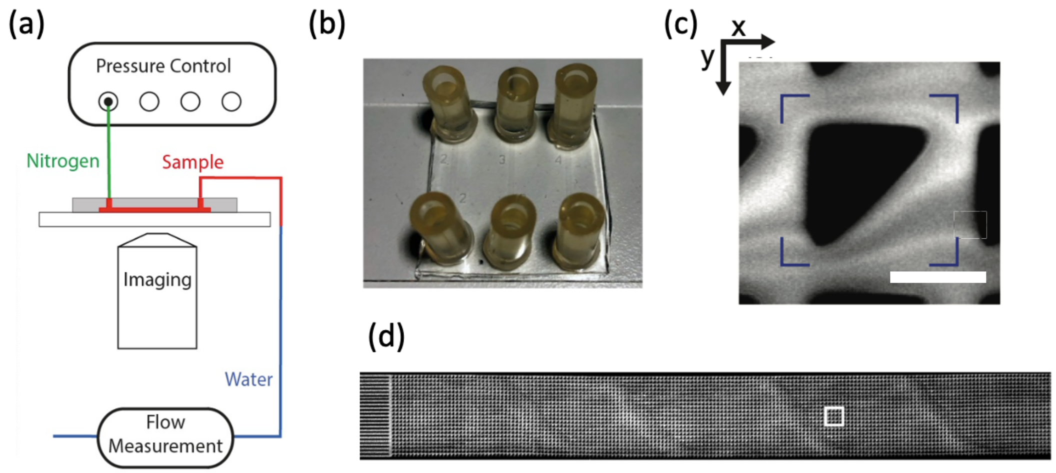

FIG. S3. Overview of the experimental setup. (a) Flow is created in the device using pressurized nitrogen (green). The sample (red) flows through the device and is in contact with water (blue) approximately a cm after the outlet. The tubing, filled with water, continues approximately 30 cm before it is connected to the flow meter. This minimizes any errors in readout due to the sample since the flow meter has been calibrated for water. (b) Several devices are fabricated in parallel for ease of experimental replication. Tubes are attached as fluid reservoirs and for connection to pressure control and flow measurement devices. (c) Image of individual pillar. The blue lines indicate the ROI for the acquisition of intensity data for Fig. 4, Fig. S10, and Fig. S11. Scale bar is 20 μm. (d) Example of a fluorescent low-magnification micrograph where waves are observed. The white square indicates the approximate location of the high magnification micrographs presented in Fig. 3, and where the intensity data was acquired as described in (c).

2. DEVICE MICROFABRICATION

PDMS devices were cast on molds fabricated in mrDWL40 resist (Micro Resist Technology GmbH, Berlin, Germany). Four-inch wafers were treated for 60 s with oxygen plasma (Plasma Preen, NJ, USA) and resist was spun at 5000 rpm for 60 s to a thickness of 13.3 μm. The design, which was made with the layout editing software L-Edit 16.02 (Tanner Research, Monrovia, CA, USA) was exposed at approximately 450 mJ/cm^2 with a 405 nm laser using an MLA150 maskless lithography system (Heidelberg Instruments GmbH, Heidelberg, Germany). After hard baking at 120°C for 10 min, the resist layer settled at 11.5 μm. A thin layer of aluminium oxide followed by a monolayer of Perfluorodecyltrichlorosilane (FDTS) was deposited in an Atomic Layer Deposition (ALD) system (Fiji – Plasma Enhanced ALD, Veeco, NY, USA), to assist with demolding. The cast PDMS pieces were cut to size and bonded to microscope slides via surface activation of both glass slides (60 s) and PDMS (10 s) with air plasma (Zepto, Diener electronic GmbH & Co. KG, Ebhausen, Germany). Holes were punched through the PDMS and 3 $\times$ 5 mm (inner $\times$ outer diameter) silicone tubes of 1 cm length were glued to the PDMS to function as both liquid reservoirs and connections for the application of pressurized nitrogen to drive flow.

3. EXPERIMENTAL SETUP AND DATA ACQUISITION

An overview of the experimental setup is provided in Fig. S3. The flow was generated by applying nitrogen gas overpressure, controlled with an MFCS-4C pressure controller (Fluigent, Paris, France). The flow rate was measured using a flow sensor (Flow rate platform with flow unit S, Fluigent, Paris, France) that was connected to the outlet tubing. The devices were imaged using an Eclipse Ti microscope (Nikon Corporation, Tokyo, Japan) and illuminated with an LED lamp (SOLA Light Engine, 6-LCR-SB, Lumencor Inc, Beaverton, OR, USA).

Objectives with magnifications 2$\times$ (Nikon Plan UW, NA 0.06), 20$\times$ (Nikon CFI Plan Apochromat λ, NA 0.75), and 40$\times$ (Nikon Plan Fluor, NA 0.60) were used with a Scientific CMOS camera (Hamamatsu Orca Flash 4.0, Hamamatsu, Shizuoka Pref., Japan, pixel sizes of 6.5 μm $\times$ 6.5 μm). The videos were recorded with frame rates in the range 10 to 33/s depending on applied pressures.

For the two-color imaging we used a Optosplit II (Cairn Research Ltd., Faversham, UK). For these measurements we used a 4x objective (Nikon Plan Apo λ, NA 0.2).

The image processing took place using our own custom made Python code using standard packages.

Data was acquired on different scales ranging from whole devices down to individual pillars. For time series of the

intensity (correspondiong to DNA concentration) around one pillar, Fig. S3(c) shows the area for which the pixel values were recorded. For movies of a small number of pillars, the white square in Fig. S3(d) shows the region of interest for which the data was acquired.

4. CALCULATIONS OF REYNOLDS NUMBERS

Dimensionless numbers are widely used in microfluidics. It is essential to be conscious that they can differ in the devices we use, both in terms of space and time. The main goal is to give a general understanding of the nominal characteristics of the fluids and the factors that affect the behavior of the fluids in the devices. The Reynolds number, Re [Eq. (S1)], is used to illustrate the connection between inertial forces and viscous forces, and is calculated using the equation:

$$Re = \frac{\rho u G}{\mu}, \tag{S1}$$

where u is the average fluid velocity in the gaps between the pillars, G is the gap between the pillars, ρ is the density of water and μ is the viscosity. The mean flow velocity, u, is calculated by dividing the mean measured volumetric flow rate, Q with the cross-sectional area of the device, A, as $u = Q/A$. The cross-sectional area is calculated as $A = h \cdot G \cdot N_{gaps}$, where $h = 11.5$ μm is the channel depth, then by taking as an example device 1, $G = 9$ μm is the gap width (perpendicular to the flow) at the bottom of the triangular pillars (see Fig. S1(b)), and $N_{gaps} = 22$ is the number of gaps.

In our case, the Reynolds numbers are always much lower than 1, so that we can neglect any inertial effects.

To estimate the viscosity, μ, of the solutions containing the DNA, for the calculation of Re we compared the flow rate at a pressure difference of 40 mbar through one device with a pillar array with just water with the value of the flow rate with our DNA solution. The measured flow rates were for water 0.17 μL/min and for the DNA solution 0.013 μL/min. Thus the viscosity of the DNA solution at this flow rate is 13 times that of water. With a temperature of the solutions of 20 ℃, 1.00×10^{-3} Pa s. We thus estimate the viscosity for the DNA $\mu_{DNA} \approx 13 \times 10^{-3}$ Pa s.

5. SAMPLE PREPARATION

Experiments took place with high concentration (400 ng/μL) of long DNA (λ-phage). The DNA was stained with the green bisintercalating dye YOYO-1 at a DNA basepair to dye ratio of 200:1 for the symmetry experiments with device 1. For the wave-suppression studies with device 2, YOYO-1 was used at a basepair to dye ratio of 50:1 and with the red bisintercalating dye YOYO-3 at a basepair to dye ratio of 25:1. For the mixing studies with device 3, YOYO-1 and YOYO-3 were used at a basepair to dye ratio of 50:1. The DNA solutions were based on $\approx 5\times$ Tris ethylenediaminetetraacetic acid (EDTA), (TE)-buffer, and 0.1% Pluronic.

6. OPTICAL ARTEFACTS

We characterized optical artefacts in the forms of stripes that resemble waves in empty devices. We find that in contrast to the waves, these patterns do not move. Since the PDMS devices are empty, the patterns do not originate in any elastic turbulence, see Fig. S4, and instead originate in an optical effect most probably due to the interaction of the excitation light and the device array. Depending on the angle of incidence of the illuminating light, these stripes vary in intensity and can either be positive or negative. We observe these stripes to always have the same angle and wavelength relative to the long axis of the array. One clear example of these types of artefacts can be found in some of the (2× objective) micrographs, Fig. S5. The wave-like artefact located to the right in the images remains unchanged for all snapshots.

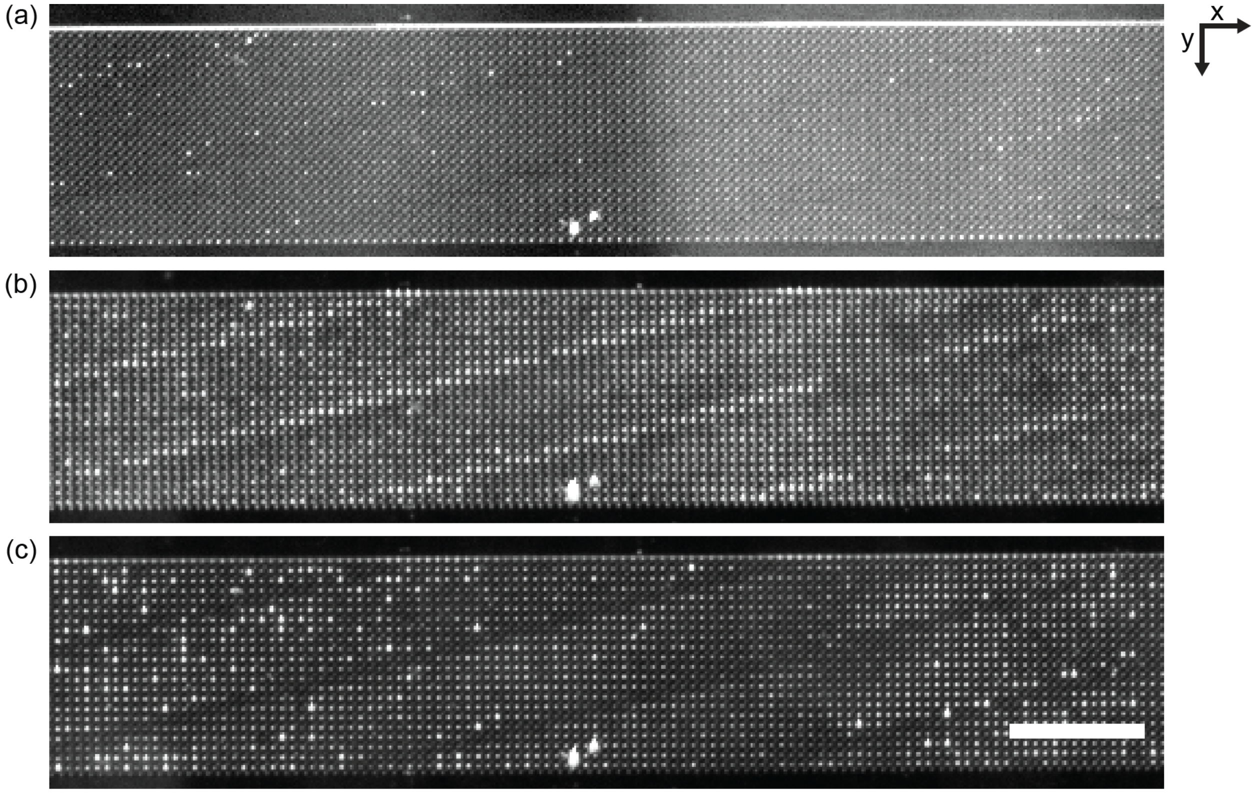

FIG. S4. Brightfield micrographs of the array in device 1 lacking any sample. (a) Illumination from top-side of the array. (b and c) Illumination from two separate side-way directions. Optical artefacts are visible as either positive (b) or negative (c) oblique stripes. Scale bar is 500 μm.

7. WAVES FOR A WIDE RANGE OF FLOW VELOCITIES

To provide a broader view of the behavior of the waves, we present additional data for a wider range of flow velocities than shown in Fig. 1 in the main text, see Figs. S5 and S6.

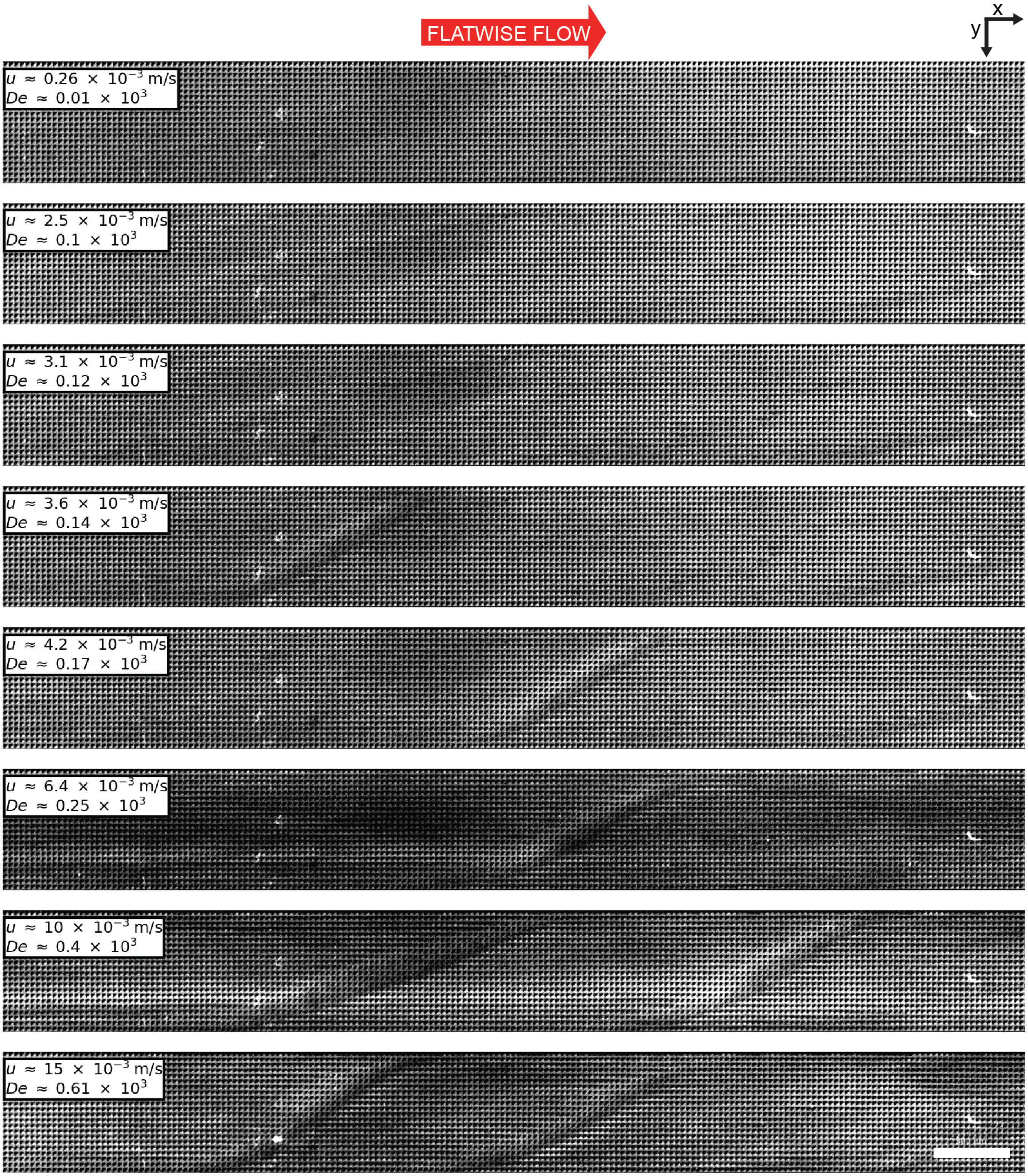

FIG. S5. Flatwise DNA flow - fluorescent micrograph snapshots at low magnification ($2\times$) for multiple flow velocities in device 1. While these data represent the behavior at several fixed applied pressures, the dynamics of the flow for a slowly oscillating applied pressure is seen in the Supplemental Movies S4 and S5. The brightness and contrast settings have been adjusted to be the same for all micrographs. Scale bar is 500 μm.

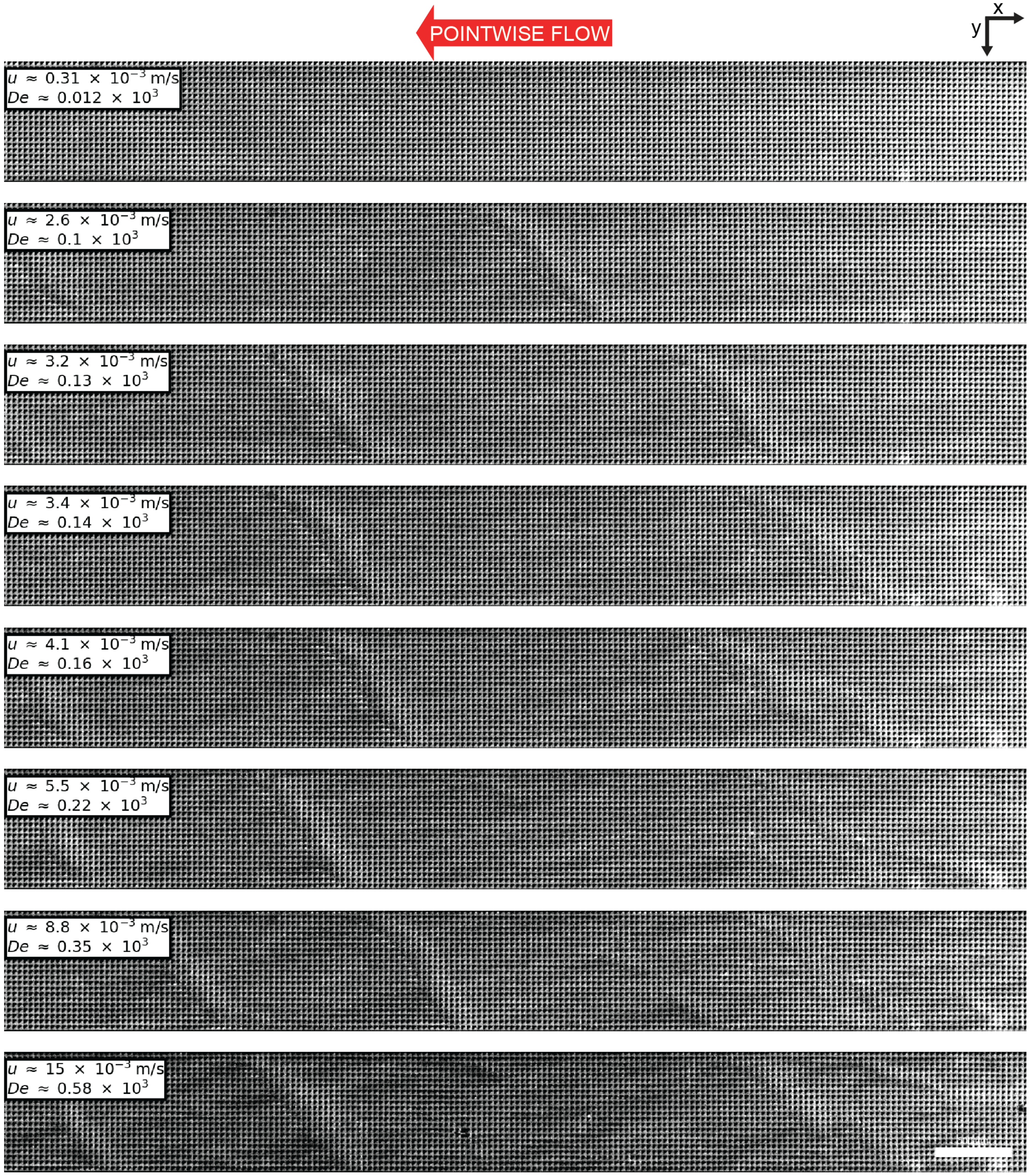

FIG. S6. Pointwise DNA flow - fluorescent micrograph snapshots at low magnification (2×) for multiple flow velocities in device 1. While these data represent the behavior at several fixed applied pressures, the dynamics of the flow for a slowly oscillating applied pressure is seen in Supplemental Movies S4 and S5. The brightness and contrast settings have been adjusted to be the same for all micrographs. Scale bar is 500 μm.

8. FREQUENCY ANALYSIS

8.1. Image Pre-processing

A number of pre-processing steps are performed before the images are analyzed. There are defects on the camera sensor and the illumination is not fully spatially homogeneous. This background is subtracted by inversely multiplying the image data with a median z-projection of a homogeneous fluorescent image. The images are then rotated so that triangular pillars point rightwards and so that the array is aligned with the image axes. The average background signal of the non-fluorescent microchannel area outside of the channels is then subtracted from the image. The image is then cropped so that it is analyzed without the microchannel walls. The images are originally captured as 16-bit. To speed up the analysis, the images are converted from 16-bit to 8-bit. The conversion is performed with the same pixel value limits for the entire data set.

8.2. Spatial 2D Frequency Analysis

Python package NumPy's fft.fft2 function is used to create a discrete two-dimensional (2D) Fast Fourier Transform (FFT) for every frame of each video (corresponding to one pressure value or mean flow velocity) of each data set. The transform is then shifted so that the zero frequency is in the center of the two-dimensional array with Numpy's fftshift function. See Fig. S7 for a typical spectrum from a low magnification (2× objective) micrograph together with various masks to demonstrate what the different parts of the frequency spectrum represent in the spatial dimension.

To complement the normalized data shown in Fig. 2, Fig. S8 and Fig. S9 show mean Fourier amplitude spectra without further processing of video data of the same type as is given in Fig. S5 and Fig. S6. Each spectrum represents an average over FFT spectra for individual frames in each videograph. The logarithm of the absolute values of the amplitude are plotted. The panels show only the positive wavelengths in the y-direction (perpendicular to the flow direction) and not the superfluous negative ones. The high-magnitude at infinite wavelength corresponds to the zero frequency component. For all 2× videographs that were analyzed by Fourier transforms, an exposure time corresponding to 50 fps was used. For the lowest flow velocity, 0.3 mm/s the videographs were 500 frames or 100 s long. For flow velocities 3 mm/s, 4 mm/s, 6 mm/s, 10 mm/s, 1500 frames were used with 6 min, 2 min, 2 min and 2 min durations, respectively.

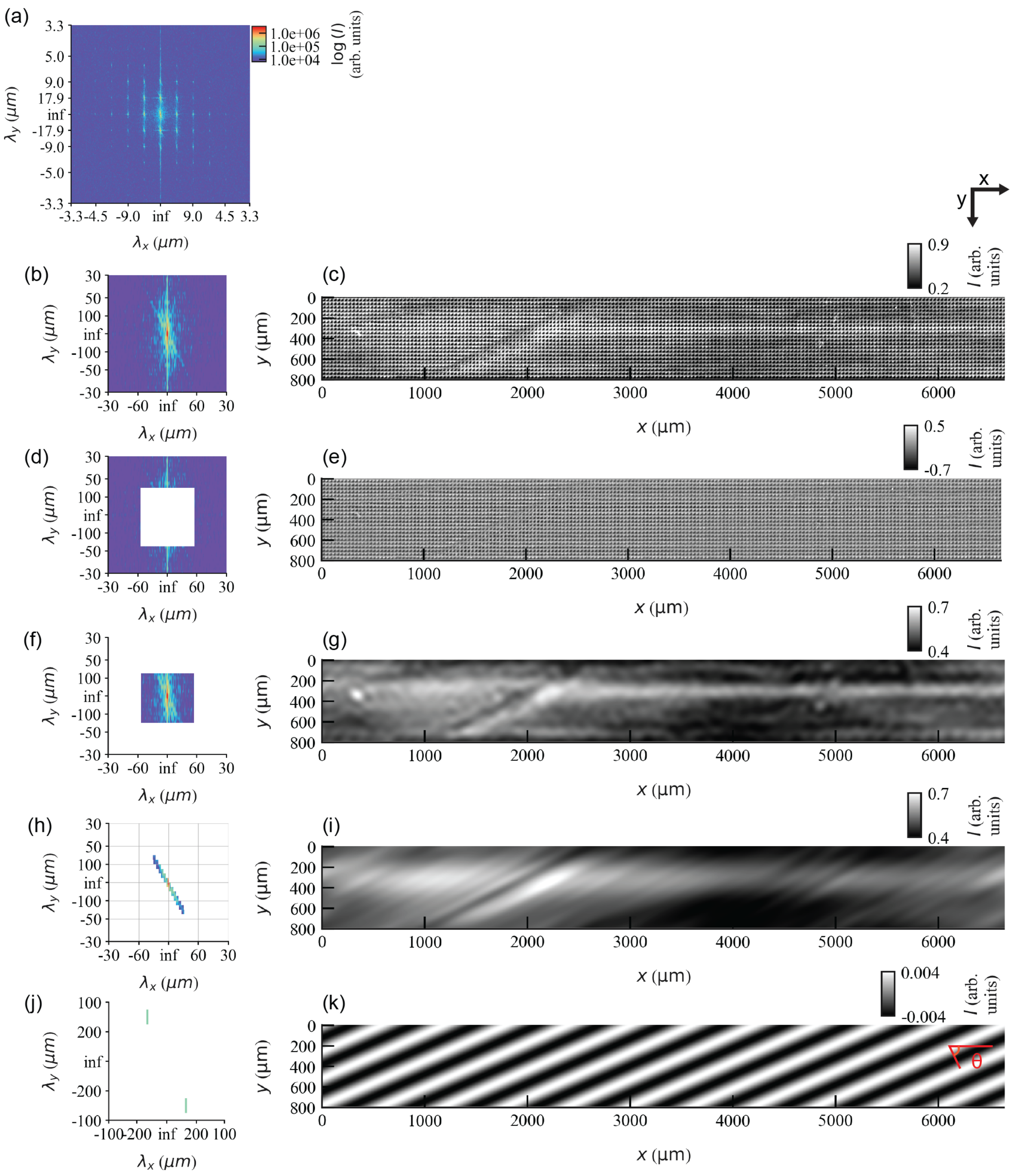

FIG. S7. Full width Fast Fourier transform (FFT) two-dimensional amplitude spectrum (a) together with frequency mask examples (b, d, f, h and j) and their corresponding inverse transforms (c, e, g, i and k), respectively. The white regions of the masks are set to zero amplitude. All spectra are based on a low magnification (2× objective) micrograph (c) with flatwise flow at $u \approx 10\,\text{mm/s}$ ($De \approx 400$) in device 1. Both the negative and positive frequency components are shown in the spectra. (b) Low frequency spectrum (zoomed-in version of (a)). (d) Square-shaped high-pass filter ($\lambda_{\text{x,y cut off}} = 60\ \mu$m). The rectangular shape is chosen due to the image basis for the FFT is rectangular and not square which would have made it easy to generate a circular shape. (f) Square-shaped low-pass filter (inverse of (d)). (h) Mask of the long wavelength region corresponding to the oblique line in the spectrum. (j) Mask of the frequency peak in in the long wavelength (low frequency) region with zero frequencies excluded. The peak has a wavelength of 121 μm and angle, $\theta = 66.5^\circ$ (see drawing in (k)). The color bar in (a) is also applicable to (b, d, f, h and j). The intensity color bar in (c, e, g, i and k) is relative to the fluorescence intensity in (c).

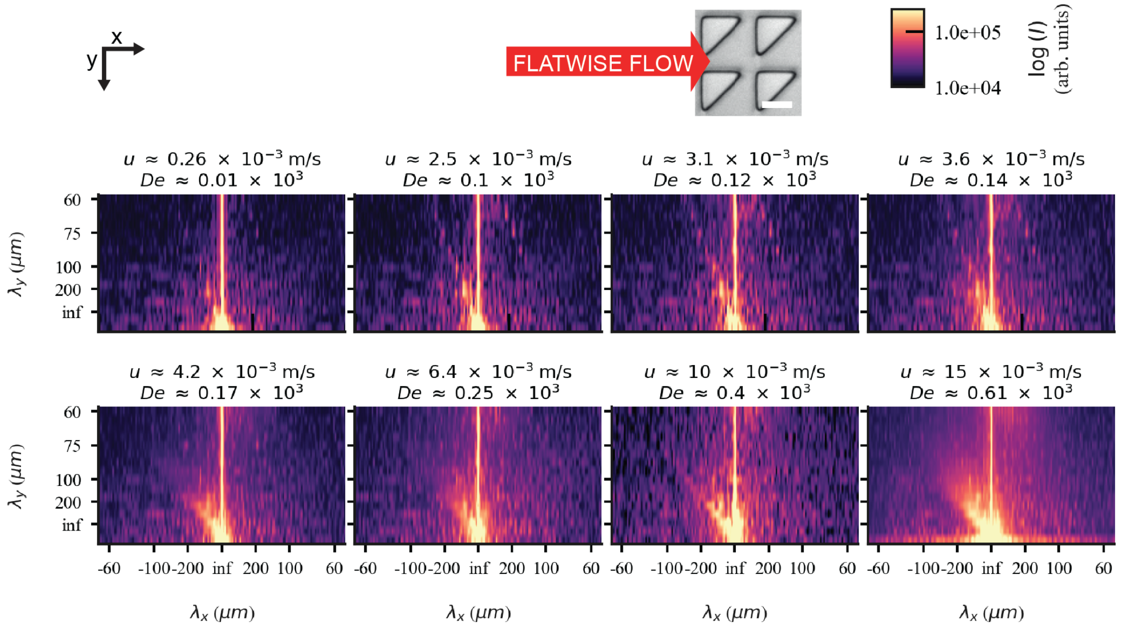

FIG. S8. Flatwise DNA flow - logarithmically scaled and time-averaged two-dimensional and long wavelength (low frequency) region of Fourier amplitude spectra. Only the positive y-frequencies are shown. The data is based on long fluorescence videos captured at a low magnification (2× objective) in device 1 with 500, 1500, 1500, 1500, 1500, 1500, 1500, and 1500 number of frames and 1.5 min, 5 min, 5 min, 5 min, 2 min, 2 min and 2 min for the flow velocities from top to bottom, respectively. The image brightness and contrast has been set so that the absolute amplitude limits are the same for all pressure values. The direction of the waves relative to the posts is indicated by insets representing a typical device used for the experiments in this work. Scale bar is 20 μm.

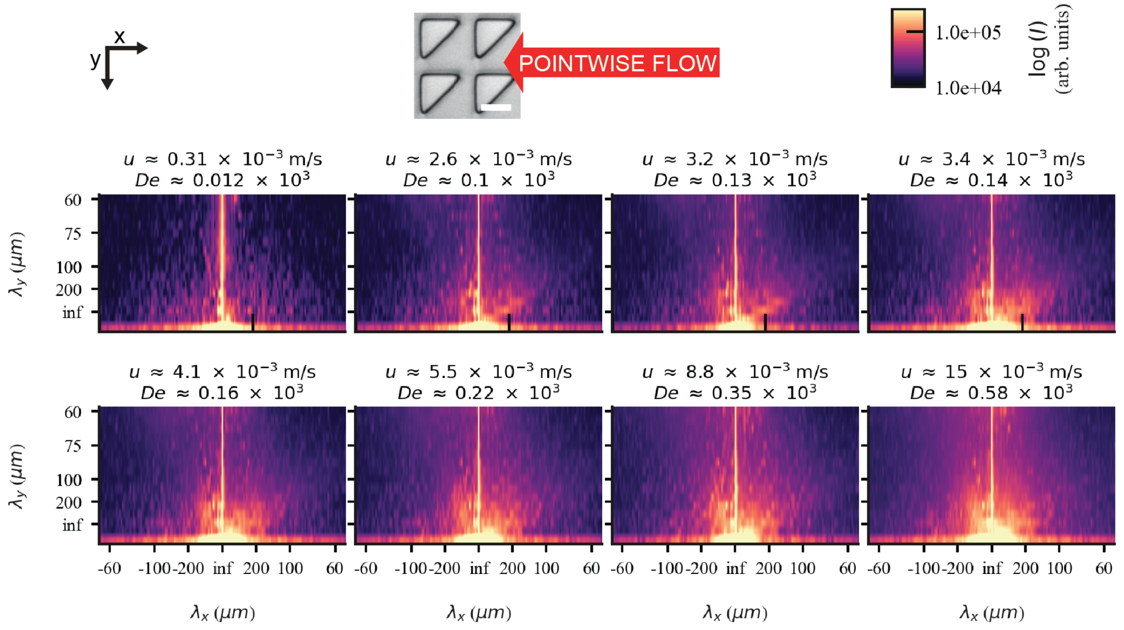

FIG. S9. Pointwise DNA flow - logarithmically scaled and time-averaged two-dimensional and long wavelength (low frequency) region of Fourier amplitude spectra. Only the positive y-frequencies are shown. The data is based on long fluorescence videos captured at a low magnification (2× objective) in device 1 with 500, 1500, 1500, 1500, 1500, 1500, 1500, and 1500 number of frames and 1.5 min, 5 min, 5 min, 5 min, 2 min, 2 min and 2 min for the flow velocities from top to bottom, respectively. The image brightness and contrast has been set so that the absolute amplitude limits are the same for all pressure values. The direction of the waves relative to the posts is indicated by insets representing a typical device used for the experiments in this work. Scale bar is 20 μm.

8.3. Temporal frequency analysis

A one-dimensional temporal (1D) Fast Fourier Transform (FFT) function (Python Package Scipy's fft.rfft) has been applied on the mean intensity signal from 20 unit cells from either flow direction. See the intensity signals in Fig. S10. The mean Fourier amplitude spectra are presented in Fig. S11.

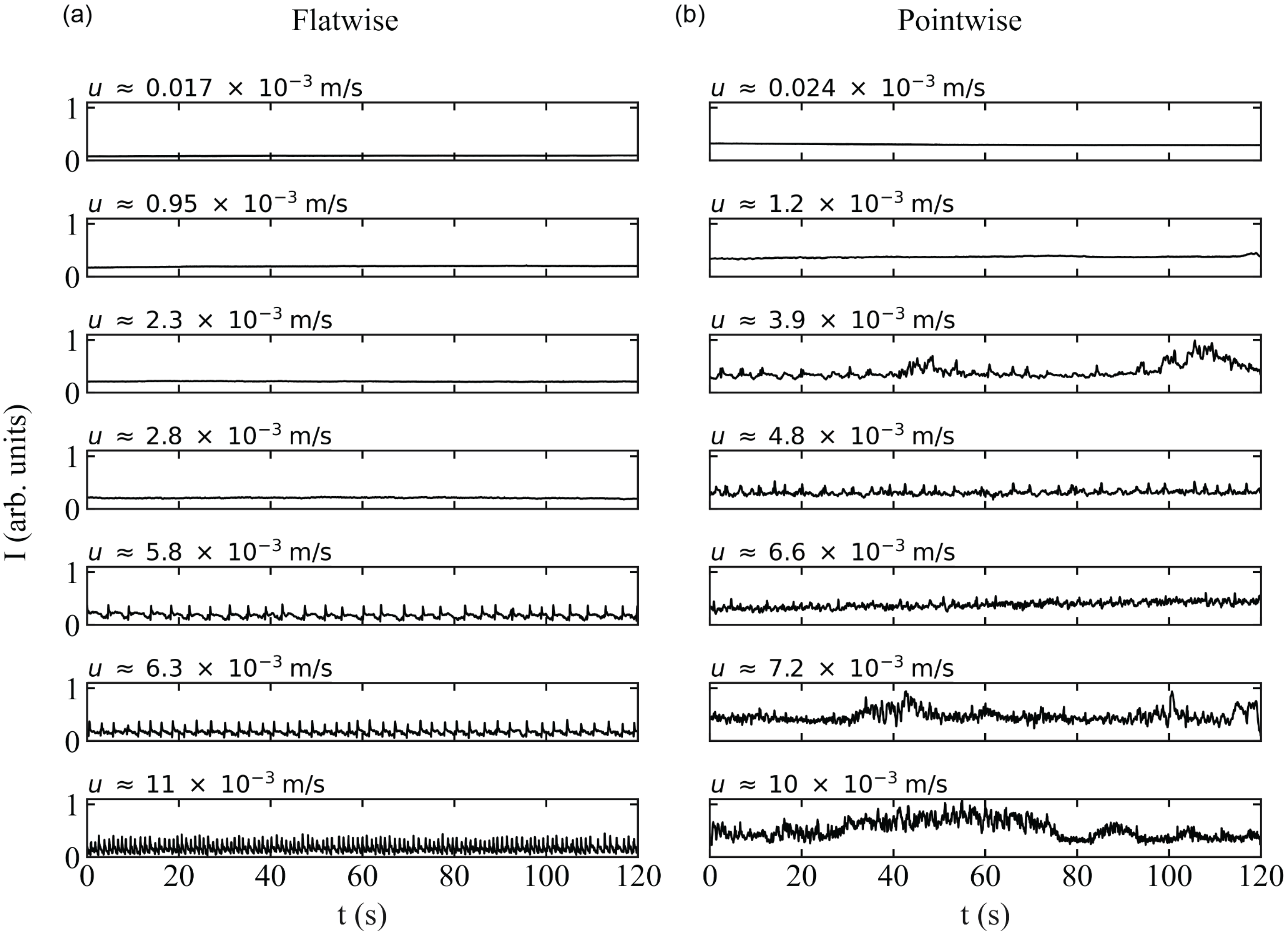

FIG. S10. Normalized time signals of the same unit cell (captured at 20× magnification) for different mean flow velocities (indicated above each graph) in device 1. See Fig. S3(c) for how the unit cell is spatially defined and Fig. S3(d) for the location of the unit cell in the device.

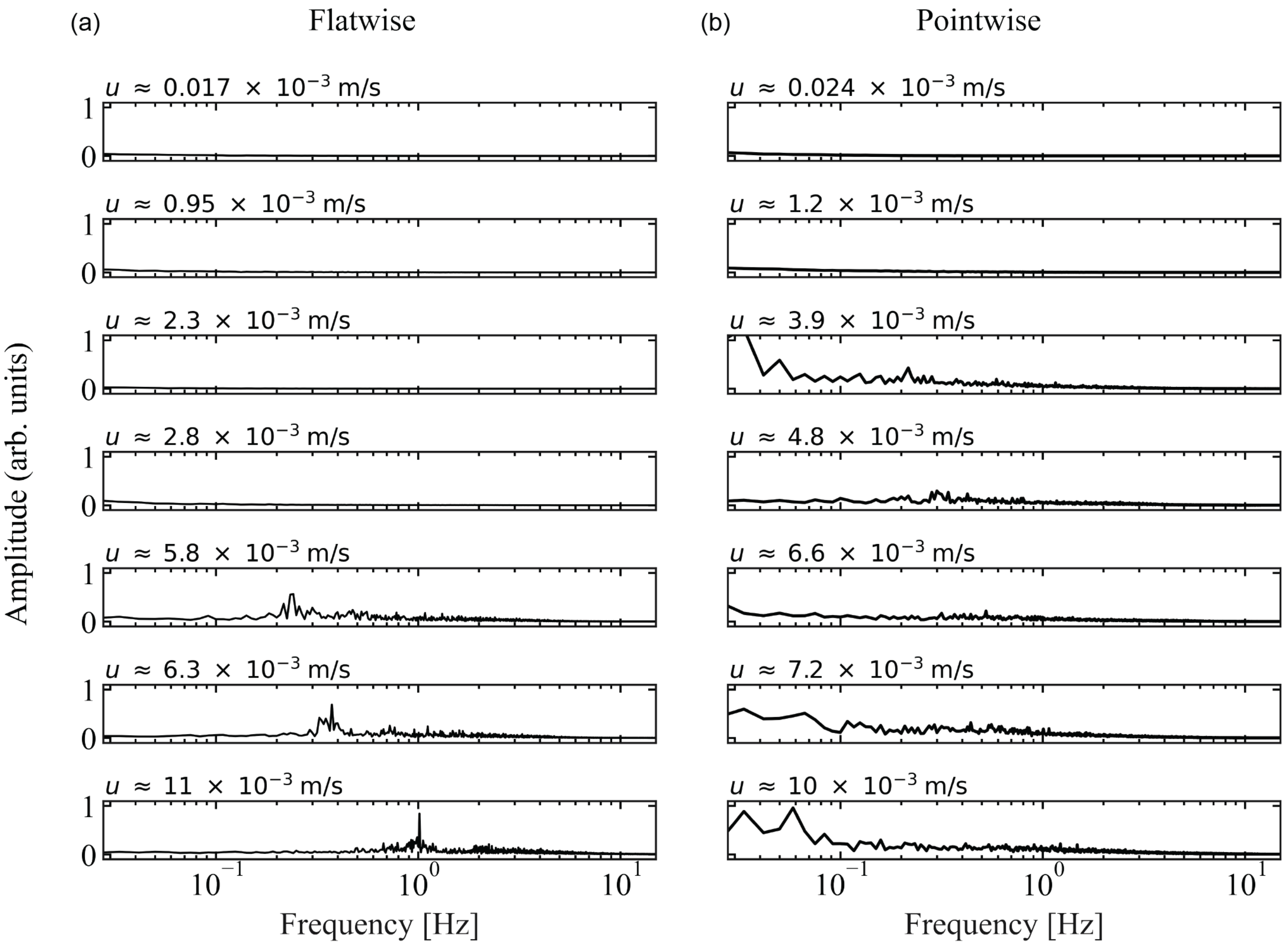

FIG. S11. Normalized and mean temporal 1D Fourier amplitude spectra of flatwise (a) and pointwise (b) flow directions for different mean flow velocities as given above each spectrum. Each presented spectrum represents the average of Fourier spectra calculated for 20 unit-cell time signals (captured at 20× magnification and duration of 120 s) in device 1. See examples of individual time signals in Fig. S10.

9. MOVIES

Supplemental Movie S1. Overview of wave formation in low-magnification (2× objective) fluorescent videographs at high flow velocity for pointwise ($u \approx 8.7\,$mm/s, $De \approx 350$) and flatwise ($u \approx 10\,$mm/s, $De \approx 400$) flows in device 1. The movie corresponds to the type of data shown in Fig. 1.

Supplemental Movie S2. Flow patterns around pillars in two high-magnification (40× objective) fluorescent videographs side by side depicting pointwise and flatwise flows respectively in device 1 at flow velocities corresponding to the data shown in Fig. 3.

Supplemental Movie S3. Flow patterns around pillars in slowed-down high-magnification (40× objective) fluorescent videographs at high flow velocity, first for pointwise flow (6.3 mm/s, $De \approx 250$), followed by flatwise flows (6.6 mm/s, $De \approx 260$) in device 1.

Supplemental Movie S4. Overview of wave formation as a function of flow rate in low-magnification (2× objective) fluorescent videograph where the applied pressure difference is slowly cycled between −200 mbar and 200 mbar in device 1. In this range waves are observable only for the pointwise flow direction. The peak flow velocity corresponds to approximately 2–4 mm/s. The size of the white bar indicates the relative pressure difference. The

movie corresponds to the type of data shown in Fig. 1.

Supplemental Movie S5. Overview of wave formation as a function of flow rate in low-magnification (2× objective) fluorescent videograph where the applied pressure difference is slowly cycled between −1000 mbar and 1000 mbar in device 1. In this range waves are visible for both flow directions. The peak flow velocity corresponds to approximately 10 mm/s. The size of the white bar indicates the relative pressure difference. The movie corresponds to the type of data shown in Fig. 1.

Supplemental Movie S6. Mixing and suppression of mixing at low flow rates. Low-magnification (4× objective) fluorescent videographs representing the comparison of the mixing obtained in presence of the waves and in absence of them due to suppression. For the alternating triangles array geometry (device 2), $Q \approx 0.58\,\mu\text{L/min}$, $u \approx 3.7\,\text{mm/s}$, $Re \approx 0.0029$ and $De \approx 150$. For the unidirectional triangles array geometry (device 3), $Q \approx 0.59\,\mu\text{L/min}$, $u \approx 3.1\,\text{mm/s}$, $Re \approx 0.0029$ and $De \approx 120$. The raw videos have been acquired at 20 fps and the videos obtained after processing, here presented, are reproduced at 10 fps for ease of visualization of the relevant features. The movie corresponds to the type of data shown in Fig. 5(b-i).

Supplemental Movie S7. Mixing and suppression of mixing at high flow rates. Low-magnification (4× objective) fluorescent videographs representing the comparison of the mixing obtained in presence of the waves and in absence of them due to suppression. For the alternating triangles array geometry (device 2), $Q \approx 3.9\,\mu\text{L/min}$, $u \approx 25\,\text{mm/s}$, $Re \approx 0.019$ and $De \approx 1000$. For the unidirectional triangles array geometry (device 3), $Q \approx 4.0\,\mu\text{L/min}$, $u \approx 21\,\text{mm/s}$, $Re \approx 0.020$ and $De \approx 850$. The raw videos have been acquired at 20 fps and the videos obtained after processing, here presented, are reproduced at 10 fps for ease of visualization of the relevant features. The movie corresponds to the type of data shown in Fig. 6(a-h).
